# Differences in the faecal microbiome of obese and non-obese pregnant women: a matched cohort study in Sweden

**DOI:** 10.1186/s12866-025-04473-8

**Published:** 2025-11-15

**Authors:** Evangelos Patavoukas, Bangzhuo Tong, Unnur Guðnadóttir, Kyriakos Charalampous, Nele Brusselaers, Ina Schuppe-Koistinen, Lars Engstrand, Emma Fransson, Eva Wiberg-Itzel, Luisa Hugerth

**Affiliations:** 1 Department of Gynecology and Reproductive Medicine, Karolinska University Hospital, Stockholm, 14186 Sweden; 2 Department of Clinical Science and Education, Södersjukhuset, Karolinska Institutet, Stockholm, 11883 Sweden; 3 Department of Medical Biochemistry and Microbiology, Science for Life Laboratory, Uppsala University, Uppsala, 75237 Sweden; 4 Department of Women’s and Children’s Health, Karolinska Institutet, 17165 Solna, Sweden; 5 Global Health Institute, University of Antwerp, Antwerp, 2610 Belgium; 6 Department of Molecular, Tumour and Cell Biology, Karolinska Institutet, Stockholm, Sweden; 7 Department of Women’s and Children’s Health, Uppsala University, Uppsala, Sweden; 8 Department of Obstetrics and Gynecology, Södersjukhuset, Stockholm, 11883 Sweden

**Keywords:** Gut microbiome, Obesity, Pregnancy, Dysbiosis, Preeclampsia, Diabetes, Hypertension, Diversity

## Abstract

**Background:**

Differences in the gut microbiome between lean and obese individuals, even twins, have been recognized for over a decade. The causative role of the microbiome in obesity is known from both mouse and human studies. In parallel, the gut microbiome has been implicated in the most common complications of pregnancy, including preterm birth, gestational diabetes mellitus, and gestational hypertension. Despite obesity being a well-established risk factor for these complications, the composition of the gut microbiome of obese pregnant individuals has not yet been studied. Here, we have examined the differences in the faecal microbiota between lean (*n* = 746) and obese (*n* = 254) pregnant women in Sweden, at two timepoints in pregnancy.

**Results:**

The differences in the faecal microbiome of one thousand lean and obese persons persist during gestation. Obese individuals have a less diverse and less rich microbiome throughout all trimesters of pregnancy. In the first trimester, 9 species differ significantly between lean and obese individuals, and 35 in the early third trimester, after adjusting for confounders. However, only one species remained significant after further adjusting for bowel transit time, which differed significantly between lean and obese participants. Additionally, obese individuals harbored a consistently lower potential to produce propionate in their gut microbiomes, even after adjustments.

**Conclusions:**

The faecal microbiome adapts to pregnancy with some key differences between lean and obese mothers, even in late pregnancy. Crucially, there is an over-abundance of opportunistic pathogens in the microbiome of obese pregnant women in the third trimester. This may be a potential underexplored mechanism explaining the increased rates of pregnancy complications among obese patients. Diet, probiotics, or medication interventions to correct the gut microbiome of pregnant obese individuals could potentially improve their pregnancy outcomes.

**Supplementary Information:**

The online version contains supplementary material available at 10.1186/s12866-025-04473-8.

## Background

Obesity is a significant public health problem in almost all middle- and high-income countries. According to the WHO, one out of five individuals will be obese by 2025 [1]. This global rise in obesity can be attributed to an increasingly obesogenic environment (e.g., reduced energy expenditure, higher prices of fresh produce, food additives, and lower availability of healthy food [2]), but animal and human evidence also supports a role of early-life microbiome disturbances, such as antibiotic usage, in promoting obesity [3]. Generally, obesity has been associated with gut microbiome imbalance (dysbiosis), lower microbiota diversity, and reduced microbial gene richness [4]. Recent studies propose that a high-fat diet induces dysbiosis and subsequent systemic inflammation [5, 6], which may partially explain obesity-associated dysbiosis.

Pregnancy is a biological process that requires synchronized changes in the immune system, hormonal profile and weight of the woman in order to preserve the health of the mother and offspring [7]. The importance of the gut microbiota in pregnancies has been demonstrated in the last decades [8], although mostly in small cohorts and in a cross-sectional approach. Studying the maternal microbiome has implications not only for pregnancy but for the lifelong health of the neonate. A systematic review reported alterations in the maternal microbiome throughout pregnancy, resulting in elevated levels of bacterial byproducts in the mother’s bloodstream [9], including short-chain fatty acids (SCFA), amino acids, vitamin K and B-complex vitamins [10]. These products can enter the fetal circulation, leading to potential repercussions for both physical and mental health outcomes and influencing the colonization of the infant’s microbiome [9].

Inflammatory profiles change a lot during pregnancy: in the beginning, a moderate inflammatory environment is more favorable to embryo implantation, while later on, the local decidua needs to provide an anti-inflammatory and immune-resistant environment in favor of the embryo. Finally, approaching the delivery, a shift to a pro-inflammatory state is observed [11]. It is not clear whether the gut microbiome changes in parallel with these immune adaptations. One Chinese study found a gradual increase in the alpha diversity throughout pregnancy in the 55 included women [12], whereas other studies did not find any significant alterations [13] or even a decrease [14]. Most of these studies were relatively small, and their results are not conclusive.

Pregnant women with a higher Body Mass Index (BMI) are an increasing group in antenatal care worldwide and in Sweden [15]. They need to be studied more thoroughly to get better care to minimize the risk of complications during their pregnancies. Women with preconceptional and maternal obesity have higher risk of gestational complications such as early pregnancy loss, fetal malformations, large for gestational babies, preeclampsia, gestational diabetes and hypertensive disorders compared to normal weight pregnant persons [16]. Since obesity seems to change the gut microbiome and pregnancy too, it is important to understand what is happening in the microbiome in maternal obesity.

Here, we leverage the most extensive longitudinal pregnancy microbiome study to date, the SweMaMi cohort [17], to examine the variation in the gut microbiome in obese pregnant women and see if it differs from that of normal-weight pregnant women in terms of species richness, diversity, specifically altered species, and critical microbial gene functions between the second and the third trimester.

## Materials and methods

### Cohort

The Swedish Maternal Microbiome (SweMaMi) study is a prospective cohort study examining the microbiome during pregnancy, with adverse pregnancy outcomes, including preterm birth and pregnancy loss, as its primary outcomes [17]. Its study protocol has been approved by the Regional Ethics Board, Stockholm, Sweden (2017/1118-31). All participants were at least 18-years-old at enrollment and provided informed consent when completing the first online questionnaire. Participants filled in extensive questionnaires on health and pregnancy before gestational week 20 and were consequently sent microbiome collection kits for home sampling. The same procedure was repeated around gestational week 30 and approximately 5–8 weeks postpartum [17]. For this study, only samples collected at the first two timepoints (during pregnancy) were analyzed. The questionnaires in Swedish and English are available from Zenodo: 10.5281/zenodo.15369670. This study was performed according to the declaration of Helsinki.

Here, we conducted a nested case-control study comparing the faecal microbes of 254 obese pregnant women (all participants with self-reported pre-pregnancy BMI > 30 and who provided a faecal sample). These were individually matched to 746 lean controls (self-reported pre-pregnancy BMI < 25) based on the following matching criteria: parity (nulliparous or parous), gestational week of first fecal samples (± 1 week), and age (± 5 years).

### DNA extraction, sequencing and annotation

DNA was extracted, sequenced and annotated as previously reported [17]. Briefly, samples were collected at home and preserved in DNA/RNA-shield for shipment. They were then stored at −80 °C for up to 5 years before being thawed and subjected to bead-beating with ZR BashingBead lysis tube (0.1 & 0.5 mm) before having their DNA purified with a ZymoBIOMICS 96 MagBead DNA protocol. 50 ng of total DNA were prepared with the MGI’s FS library prep set and sequenced on MGI T7 instruments using paired-end 150 bp reads, yielding a median library size of 61 million reads per sample (range: 21.3–116.5.3.5 million). Samples were trimmed with fastp v.0.23.2 [18], human DNA reads were removed by annotating with kraken2 v.2.1.2 [19] on a reference containing only the human GRCh38 genome, and reads were annotated to the species level using the Metaphlan4 (mpa_vOct22_CHOCOPhlAnSGB_202212) pipeline [20] (table S1), as well as functionally annotated with the Humann2 pipeline. To derive the estimated abundance of gut metabolic modules (GMMs) [21], the gene families annotated from Humann2 [22] were regrouped to KEGG pathways using the built-in script humann_regroup_table.py in Humann2. The abundance table of the regrouped KEGG pathways was then used as input for Omixer-RPM (https://github.com/raeslab/omixer-rpm*)* [23] to estimate the abundance table of GMMs (table S2).

### Categorical and ordinal variables on baseline maternal health

Validated scales were used where possible, including the Pregnancy Unique Quantification of Emesis (PUQE) [24] for measuring nausea and vomiting, the Perceived Stress Scale (PSS-4) [25] for assessing stress, and the Edinburgh Postnatal Depression Scale (EPDS) [26] for evaluating mental distress. Additionally, some variables used were standard to all SweMaMi analyses, but not validated. These include:

- Bowel transit time. Based on the question “How do your stools typically look like?” and the Bristol stool scale (1–7) [27]. Regular transit (only 3–4), slow transit (only 1–4), fast transit (only 3–7), varied transit (both 1–2 and 5–7). This variable was calculated separately at each time-point.
- Socioeconomic score: one point each was given if having a bachelor’s degree or higher, working full-time, and married/cohabiting with partner.
- Diet score: five-point scale, with one point each given for fruits, vegetables or whole grain bread daily and two possible additional points for sweetened or sugar free beverages once a week or more seldom. This variable was calculated separately at each time-point.
- Daily fiber: daily consumption of at least one of fruits, vegetables or whole grain bread. This variable was calculated separately at each time-point.

### Descriptive statistics

These statistical analyses were performed using IBM SPSS Statistics version 29.0.2.0 (IBM Corp., Armonk, NY, USA). Continuous variables were presented as means and standard deviations (SD), and categorical variables as frequencies and percentages. Unpaired t-tests were used to compare continuous variables and Mann-Whitney U-test for categorical variables between the obese and lean groups. A *p*-value of < 0.05 was considered statistically significant.

### Biostatistics

All further analyses were conducted in the R programming environment (R version 4.3.3), using libraries “vegan_2.6-4” [28], “ggpubr_0.6.0”, “ggplot2_3.5.1” [29], “dplyr_1.1.4” [30], “tidyverse_2.0.0” [31], “phyloseq_1.46.0” [32], “microbiome_1.24.0” [33], “ape_5.8” [28, 34] and “maaslin3_0.99.16” [35].

Alpha-diversity was measured by observed species richness, as well as Shannon’s entropy and Pielou’s evenness using relative abundance from MetaPhlan4. Two-sample t-tests were used to assess the statistical significance of the differences in alpha-diversity metrics between the lean and obese groups within each time point. Linear regression was used to explore the correlation between BMI score and alpha diversity.

The distance between samples was calculated using either Aitchison’s or Jaccard’s distances based on the relative abundance. Mann–Whitney U test was used to compare the within-individual distances between time points. Univariable PERMANOVA were computed using the function adonis2 from package “vegan”. The variables with the strongest and significant variables in adonis2 that were not confounded by overdispersion (function betadisper) were selected for further analysis in multivariable models using a stepwise forward selection approach, adding variables in decreasing R² order until they were no longer significant. Selected covariates were also used in the differential abundance models.

The differential abundance of bacterial species and gut metabolic modules (GMMs) was calculated using MaAsLin3 separately for each time-point. The relative abundance of bacterial species was passed as input of MaAsLin3 with Total-Sum-Scaling (TSS) and log transformation. To account for the abundances of microbial genes not mapped to GMMS, an additional feature labeled “Others” was introduced for each sample to guarantee a total sum of 1 million and quantification of features as Counts Per Million (CPM). The GMM abundances were then normalized by TSS and log transformed and analyzed with MaAsLin3. These analyses were performed in three different models:

1. Crude model, where obese or not (lean/obese) is exposure and total number of host-removed reads is covariate.
2. Adjusted multivariable model 1, where obese or not (lean/obese) is exposure and total number of host-removed reads, socioeconomic score (0–3), age (in years), and diet score (0–4) are covariates.
3. Adjusted multivariable model 2, where obese or not (lean/obese) is exposure and total number of host-removed reads, socioeconomic score (0–3), age (in years), diet score (0–4) and colonic transit time as −1 (slow transit), 0 (regular transit) and + 1 (fast transit) are covariates. Subjects with “various” transit times were excluded from this sub-analysis.

In all three models, the total number of host-removed reads was also included as covariate to increase the reliability of prevalence association. To increase the interpretability of MaAsLin3 result, features that are assigned with “error” were removed; features that are significantly associated with group of interest by prevalence were removed if N_not_zero is larger than half of the sample size; features that are significantly associated with group of interest by abundance were removed if N_not_zero is larger than half of the sample size. Species and modules with a BH-adjusted *p*-value less than 0.05 and a coefficient larger than 0.5 were considered significantly different. Additionally, MaAsLin3 was also applied to explore the change of species across pregnancy by fitting time point as exposure, total number of host-removed reads as covariate and subject ID as random effect in the model.

## Results

### Cohort description

**Fig. 1:**
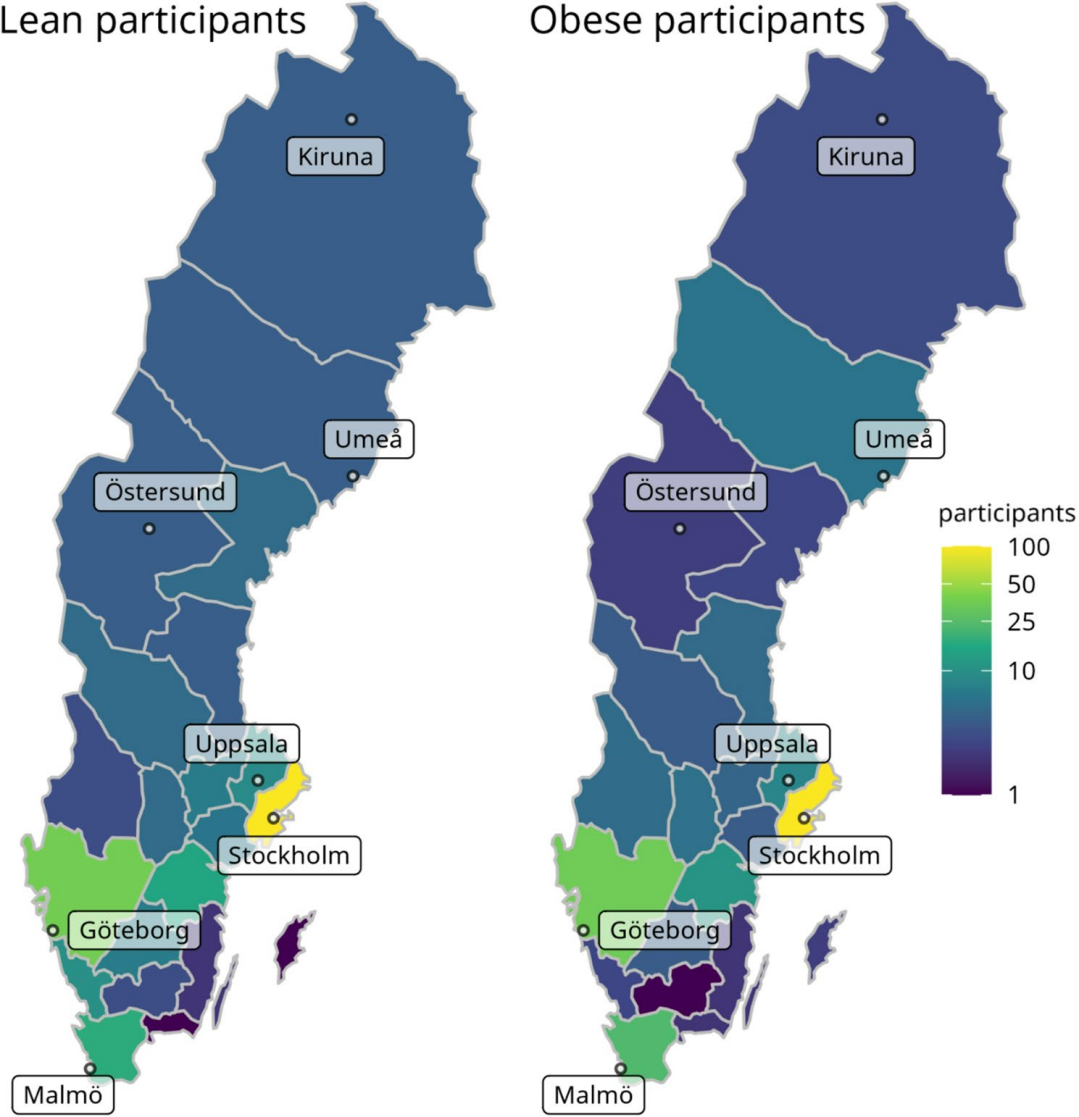
Geographical distribution of lean and obese participants, in absolute counts. Colors are given on a log scale to facilitate the visualization of less densely populated regions

Participants were recruited from all Swedish regions **(**Fig. 1**)**. The median BMI in our lean group (*n* = 746) was 22 kg/m² (IQR: 19–24 kg/m²), compared to 33 kg/m² in the obese group (*n* = 254) **(**Table 1**)**. The mean age was 32 years in both groups. Some differences were observed in alcohol consumption (more common in the lean group) and higher intake of probiotics, fibers, and vitamins in the lean group, whereas a higher proportion of polycystic ovary syndrome (PCOS), use of antidepressants, and animal contact was observed in the obese group **(**Table 1**)**. In relation to outcomes of pregnancy, a higher proportion of gestational diabetes mellitus (GDM) and preeclampsia was observed in the obese group, as expected. Additionally, there were differences in bowel transit time, with obese participants more likely to have fast or irregular bowel transit at both time-points (Table 1).

**Table 1:** Main descriptive characteristics of the lean and obese groups

Timepoint	Variable	Lean *n*(%)	Obese *n*(%)	*p*-value*
Background	Age	32 (SD:4.09)	32 (SD:4.4)	0.36
	Alcohol 3 months before pregnancy	662 (88.7)	211 (83.0)	**0.025**
	Animal contact	337 (45.2)	158 (62.2)	**< 0.001**
	Antidepressant use	34 (4.6)	27 (10.7)	**< 0.001**
	Autoimmune disease	2 (0.2)	1 (0.4)	1.0
	BMI prior	21.8 (SD: 1.5)	34.0 (SD: 4.6)	**< 0.001**
	Eating Disorder	82 (11.1)	26 (10.5)	0.86
	Ever smoking	48 (6.4)	20 (7.8)	0.52
	First pregnancy	293 (39.3)	99 (38.9)	0.99
	Hypothyroidism	74 (9.9)	32 (12.6)	0.28
	Natural conception	654 (87.7)	226 (88.9)	0.65
	Previous miscarriage (1 to 2)	92 (12.3)	85 (33.4)	**< 0.001**
	Previous miscarriages (3+)	28 (3.8)	17 (6.7)	**< 0.001**
	Socioeconomic score 0	1 (0.1)	1 (0.39)	**< 0.001**
	Socioeconomic score 1	59 (7.8)	47 (18.5)	
	Socioeconomic score 2	225 (30.2)	77 (30.3)	
	Socioeconomic score 3	461 (61.7)	129 (50.8)	
Time-point 1	Pregnancy week for sample	12.08 (SD: 3.0)	11.85 (SD: 3.0)	0.70
	Antibiotic 3 months prior	49 (6.6)	24 (9.4)	0.16
	daily fiber consumption	650 (87.1)	200 (78.4)	**0.0017**
	Diet score, mean	1.68 (SD: 1.11)	2.21 (SD: 1.18)	0.36
	GI medication during pregnancy	3 (0.4)	3 (1.2)	0.35
	Bristol rate: Fast	201 (26.9)	82 (32.4)	**< 0.001**
	Bristol rate: Normal	170 (22.8)	39 (15.4)	
	Bristol rate: Slow	202 (27.11)	54 (21.3)	
	Bristol rate: Various	172 (23.1)	78 (30.8)	
Time-point 2	Pregnancy week of sample	28.22(SD:1.32	27.88(SD:2.1)	0.66
	Antibiotics during pregnancy	78 (11.9)	34 (14.5)	0.36
	Daily fiber consumption	571 (76.5)	176 (69.2)	**0.026**
	Diet score, mean	2.38(SD:1.14)	1.92(SD:1.2)	0.21
	GI medication during pregnancy	3 (0.4)	6 (2.4)	**0.013**
	Bristol rate: Fast	146 (22.3)	85 (36.1)	**< 0.001**
	Bristol rate: Normal	152 (23.2)	31 (13.2)	
	Bristol rate: Slow	206 (31.34)	45 (19.1)	
	Bristol rate: Various	143 (21.9)	69 (29.4)	
Any time in pregnancy	Probiotic consumption	128 (17.2)	27 (10.7)	**0.016**
	Alcohol during pregnancy	62 (8.3)	9 (3.5)	**0.015**
	Vegetarian diet	109 (14.6)	26 (10.3)	0.1
	Vegan diet	12 (1.6)	4 (1.6)	1.0
Pregnancy outcomes	Gestational diabetes mellitus	17 (2.2)	46 (18.1)	**< 0.001**
	Preeclampsia	31 (4.1)	23 (9.1)	**0.004**
	Preterm delivery	61 (8.1%)	20 (7.8%)	0.9

* Significant differences (*p* < 0.05) are highlighted in bold. t-tests were used for continuous variables and Mann-Whitney U-test for categorical variables

### Taxonomy and within-sample diversity

At the phylum level, a significant increase in Actinobacteria was observed from time point 1 to time point 2 for obese women (*p* = 0.019). In contrast, lean participants showed a significant decrease in Euryarchaeota (*p* = 0.024) and Verrucomicrobia (*p* < 0.001), and an increase in *Candidatus* Melainabacteria (*p* = 0.019) (fig. S1).

**Fig. 2:**
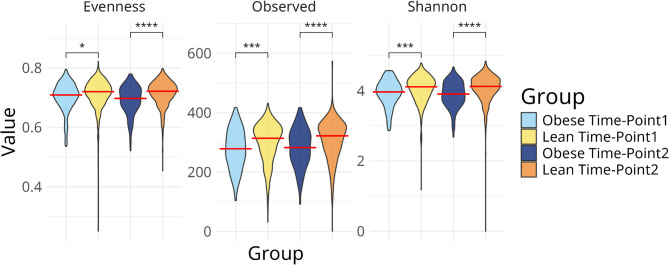
The gut microbiome of obese pregnant participants is less even, rich and diverse at both time points compared with the gut microbiome of lean participants. Comparisons were made between lean and obese participants within each time point. A red line marks the median of each distribution

Several differences in diversity were observed between the groups at the species level. Lean subjects had higher observed richness at both points (Fig. 2**)** (mean: 300.7 vs. 277.3; 308.5 vs. 282.5; both *p* < 0.0001) as well as higher diversity (Shannon’s entropy mean: 4.05 vs. 3.92; 4.07 vs. 3.89; both *p* < 0.0003) and evenness (Pielou’s evenness mean: 0.71 vs. 0.701; 0.714 vs. 0.692; both *p* < 0.05). Consistently, BMI score negatively correlated with alpha diversity (fig. S2). While both obese and lean subjects became more species-rich in the second time-point compared to the first (lean: median 318 species at TP1 vs. 323 at TP2; obese: median 290 at TP1 and 292 at TP2), this small increase was only statistically significant for lean individuals (Mann-Whitney U-test, *p* = 0.0025; fig. S3).

### Beta-diversity (between groups)

**Fig. 3:**
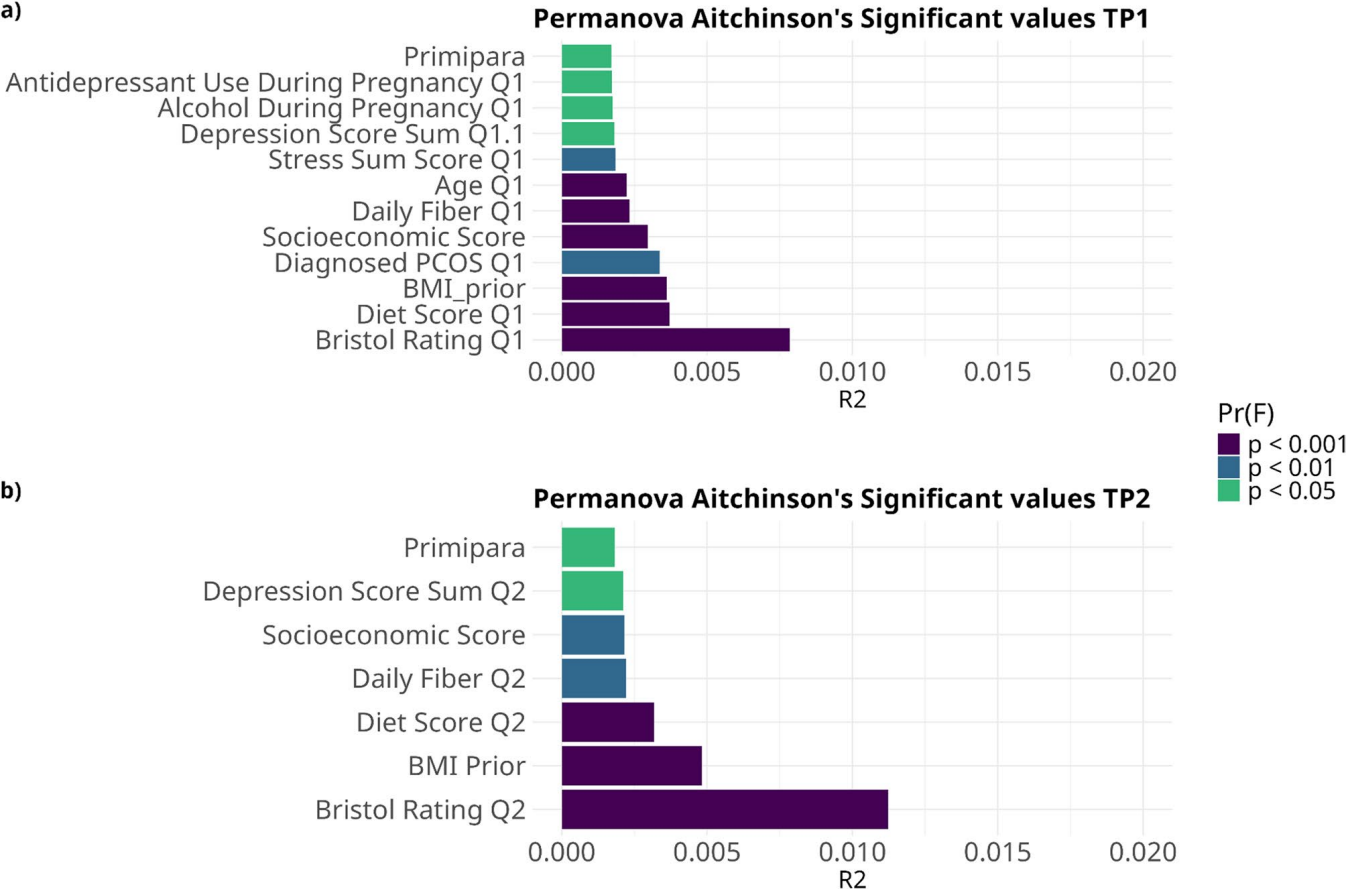
Bristol stool form scale, BMI, antibiotics and probiotics are major contributors to differences between samples at both time points. Permanova R² (length of the bar) and *p*-value (color scale) based on Aitchison distance at early and late pregnancy. Q1: questionnaire 1 (collected 1-2 weeks before sample 1). Q2: questionnaire 2 (collected 1-2 weeks before sample 2)

The distance between samples was calculated both in terms of presence/absence (Jaccard’s distance) and quantitatively (Aitchison distance). For both distance metrics and time-points, lean and obese samples were significantly different (time point 1: Aitchison’s R² = 0.003, Jaccard’s R² = 0.004; time point 2: Aitchison’s R² = 0.005, Jaccard’s R² = 0.006; all *p*-values = 0.001 on 999 permutations). However, several other characteristics, including demographics (age, socioeconomic score), diet (fiber intake, plant-based diets), and mental health (stress scores, depression scores, use of neuropsychiatric medication) were also significantly correlated with the gut microbiome at both time points **(**Fig. 3; fig. S4; table S3, S4).

### Beta-diversity (between time-points)

The distances between samples from the same subject at time point 1 and time point 2 were calculated separately for the obese and lean groups (fig. S5; S6a; S6c). Lean individuals exhibited significantly (*p* < 2.2e-16) smaller intra-individual distances (median = 19.3) compared to obese individuals (median = 20.7), indicating that their gut microbiota composition changed less over time.

### Differentially abundant species

Based on the beta-diversity analysis and previous knowledge, we decided to analyze differentially abundant species with both a crude model and two adjusted models. Both multivariable models accounted for socioeconomic score, age and diet quality, with the second model additionally considering the Bristol stool form scale as a continuous variable from slow transit (−1) to fast transit (+ 1), as described in the methods.

**Fig. 4:**
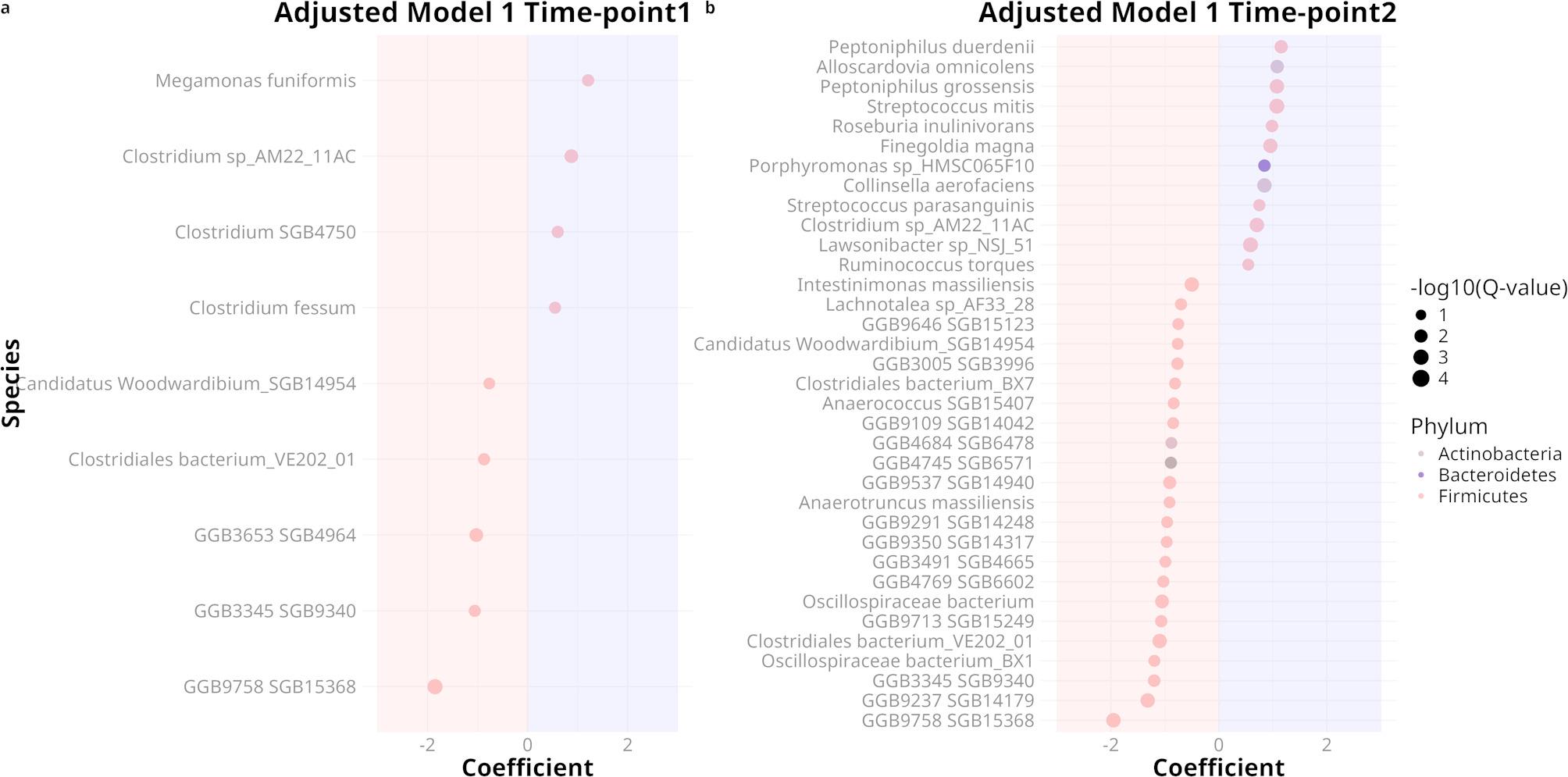
Mainly Firmicutes species exhibit differential abundance between lean and obese pregnant women in the multivariable model adjusted for socioeconomic score, age, diet score. The bubble plots display the differentially abundant species identified using MaAsLin3, with adjustments for socioeconomic score, age, diet score, and colonic transit time. The x-axis represents the coefficients of the species in the groups, while the y-axis displays the names of the differentially abundant species. Species in the blue shadow of each bubble plot were enriched in obese women, while species in the red shadow were enriched in lean women. The color of each dot indicates its phylum. (**a**) Time point 1, (**b**) Time point 2

Adjusted Model 1 revealed that 4 species were significantly enriched in obese individuals, while 5 species were significantly enriched in the lean group. In late pregnancy, these numbers increased to 12 and 23, respectively **(**Fig. 4; table S5, S6). Adjusted Model 2, which additionally accounted for Bristol stool score and excluded subjects with irregular transit (alternating fast and slow), found no enriched species in the obese group and 1 (GGB9758 SGB15368 from phylum Firmicutes) in the lean group in the early pregnancy while, in late pregnancy no significantly enriched species was found (table S7, S8). Furthermore, a total of 15 differentially abundant species was also identified to be enriched at late pregnancy compared with early pregnancy. These species collectively accounted for approximately 5% of the mean relative abundance at time point 2, whereas their mean combined abundance was close to 0% at time point 1 (fig. S7).

### Differentially abundant gut-metabolic modules

**Fig. 5:**
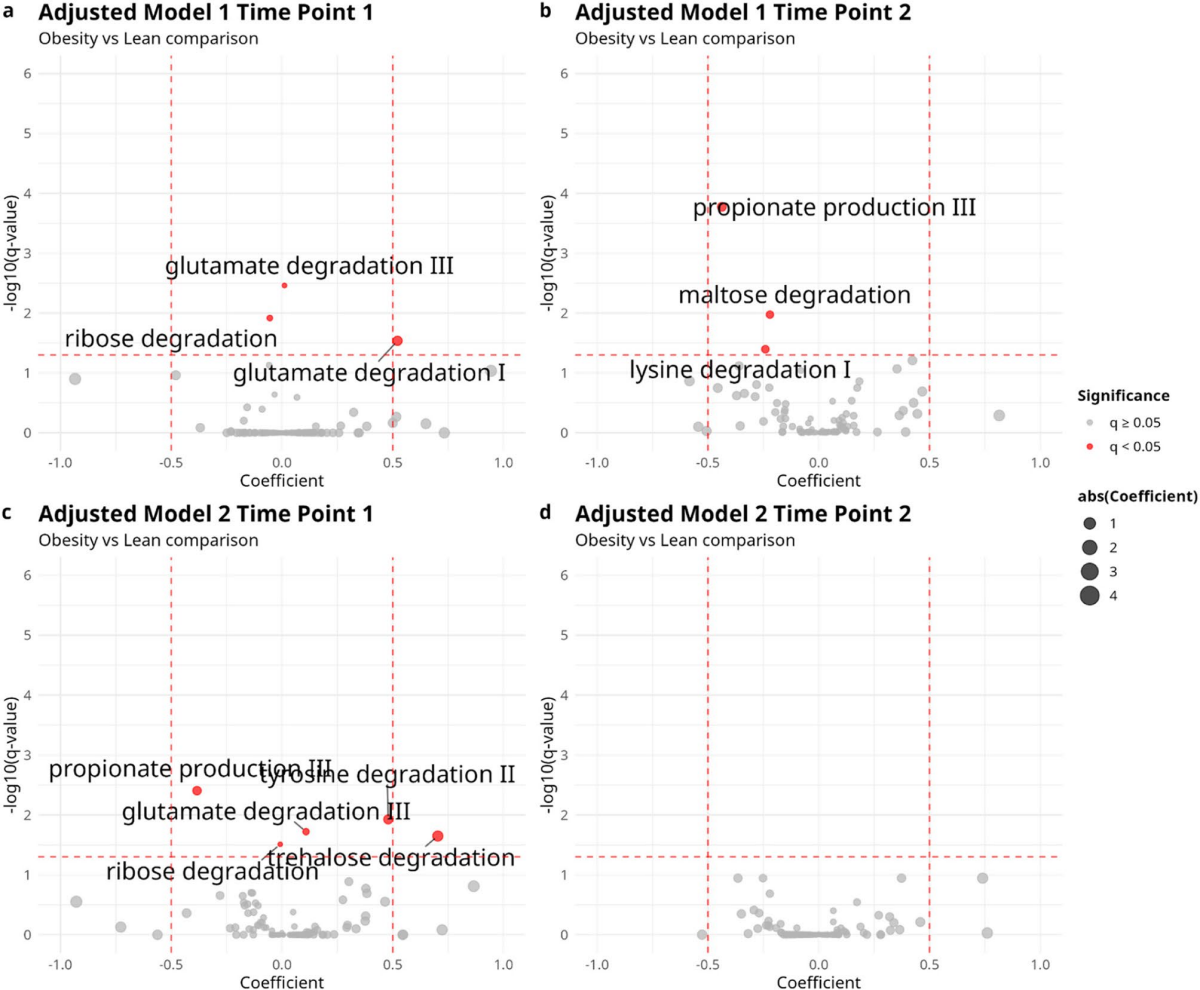
Volcano plots of the differential abundance calculated in adjusted models using MaAsLin3. Gut metabolic modules with coefficients less than 0 are more abundant in lean subjects, and those with coefficients greater than 0 are more abundant in obese individuals. Significantly abundant GMMs were highlighted in red and labeled. Bigger sizes of the data points infers higher absolute value of coefficients. The vertical red dashed line represents the threshold of the q value at 0.05, while the horizontal red dashed lines represent the thresholds of coefficients at −0.5 and 0.5, respectively. Top row: model is adjusted for sequencing depth, age, diet score, socioeconomic score and includes all participants: (**a**) time-point 1; (**b**) time-point 2. Bottom row: model is adjusted for gut transit time as well as sequencing depth, age, diet score, socioeconomic score, and participants with irregular transit time are excluded: (**c**) time point 1; (**d**) time point 2

From the sample’s total functional gene family complement, we mapped them to a subset of gene families to KEGG pathways, which are known to affect cardiometabolic health. In Adjusted Model 1, three GMMs were found to be significantly abundant at time point 1, and another three at time point 2 **(**Fig. 5a and b**)**. For Adjusted Model 2, a total of 5 GMMs were found to be significantly abundant at time point 1, and no GMM were significantly different at time point 2 **(**Fig. 5c and d**)**. Like the result of species-level differential analysis, the number of differentially abundant GMMs decreased at the late stage of pregnancy. Propionate production was the only consistent marker significantly more abundant in the lean individuals in 4 out of 6 models (tables S9 - S14).

## Discussion

The aim of this study was to characterize potential differences in the faecal microbiome between lean and obese women during the second and third trimester of gestation. Our results from this national Swedish pregnancy cohort showed that the richness of the faecal microbiome is, on average, increasing during pregnancy in the lean population, as it was also shown by Li et al. [12] - the obese group showed the same trend, but the differences were not significant, perhaps due to power. In our study beta-diversity was more stable between the two timepoints in lean women, despite both groups declaring similar reductions in their frequency of fibre consumption in the third trimester. The faecal microbiota in the lean population in our study was more similar between the second and third trimester of pregnancy while in the obese population the dissimilarities between the two time points were greater. The changes of the faecal microbiota between the two groups in the third trimester of pregnancy may imply that pregnancy itself may induce a remodeling of the microbiota in cases of obesity; however, Koren et al. also observed increased beta-diversity from the first to third trimester in a general population [14].

The overall composition of the faecal microbiome was primarily associated with large bowel transit time, diet, and BMI during the second and third trimester of pregnancy, as has been shown in other studies [36, 37]. Previous studies have shown a shift in gut microbiome composition during pregnancy toward more energy-producing communities, which are believed to be necessary for fetal growth [14]. These include Firmicutes, Proteobacteria, and Actinobacteria, where the last has significant effects on the maintenance of the gut barrier and the induction of Treg cells [38]. In our models, mostly Firmicutes species were differentially abundant between lean and obese women at each time point, which is expected in populations with a Western-style diet [39]. Those results confirm the results by Zacarias et al. [40] who found that the gut dysbiosis related to obesity was present before conception and not significantly altered during the pregnancy. We found significant differences in species composition between our groups, even after adjusting for age, primiparity, diet score, and Bristol stool form scale. The species that characterized the lean group were mostly not isolated and named (GGB, genus-level genomic bins), whereas, in the obese group, opportunistic pathogens were more abundant. Those pathogens could partially explain the higher risk for gestational complications women with obesity face. On the other hand, our obese participants presented *Roseburia inulinivorans* in higher abundance than the lean in the third trimester, which may have a compensatory roll supporting the gut barrier and producing butyrate [41].

Interestingly, species such as *Streptococcus mitis* and *S. parasanguinis*, which are linked to periodontitis, were in higher abundance in the obese group. A meta-analysis by Chopra et al. [42] showed the link of adverse pregnancy complications and periodontitis caused by *Porphyromonas gingivalis*. Periodontal microorganisms and their by-products can enter the bloodstream along with locally produced inflammatory mediators, triggering the release of acute-phase proteins. These proinflammatory substances, including cytokines, increase oxidative stress and activate Toll-like receptors (TLRs) in both maternal and fetal tissues, leading to heightened inflammation in the uterus. Additionally, microbial components may disrupt normal placental development, contributing to fetal growth restriction [42]. While our work here focuses on the fecal microbiota, there is great need for studies including different sites of the body to understand and possibly treat complications related to that by alterations of the microbiota.

We also found *Collinsella aerofaciens* to be over-represented among obese women, in agreement with Gomez-Arrango et al. [43]. They found that the presence of this species in the 16th week of pregnancy, promoting the insulin resistance during gestation, which could lead to gestational diabetes. On the other hand, *Collinsella* species are also reported to be associated with *Bifidobacterium* colonization in infants [44] and, indeed, *B. longum* and *C. aerofaciens* were found to increase from the first to the second time-point, perhaps in preparation for infant colonization.

Additionally, we examined longitudinal changes in gut microbiota composition across all participants, regardless of BMI category. A total of 15 species were significantly enriched in the third compared to the second trimester. These species, although low in abundance overall, collectively increased from near 0% at trimester 2 to approximately 5% of the mean relative abundance at trimester 3. Notably, some of these enriched taxa, including members of the *Bifidobacterium* and *Collinsella* genera, have been implicated in early-life microbiota development, where *Bifidobacteria* are known as key pioneers of the infant gut [45, 46], and *Collinsella* co-occurs with *Bifidobacterium* colonization in young infants [44]. These findings may suggest preparatory shifts in the maternal microbiota that support vertical microbial transmission to the neonate.

When analyzing gut metabolic modules, only propionate production was consistently found to be significantly more abundant in the lean subjects. Roy et al.. found that propionate and butyrate significantly reduced the inflammation-induced expression of pro-inflammatory mediators in the human placenta and adipose tissue of pregnant women [47]. They also demonstrated that propionate and butyrate significantly improved insulin sensitivity, suggesting a potential role for short-chain fatty acids in preventing and treating Gestational Diabetes Mellitus (GDM), whose risk increases by approximately 4% for every 1 kg/m² increase in BMI during pregnancy [48]. Another study showed significantly lower levels of propionate in pregnant women experiencing preeclampsia [49], whose risk doubles for every 5–7 kg/m^2^ increase in BMI [50].

Our study has several important strengths, but also some limitations that affect data collection and analysis. It is a large, well-characterized cohort of pregnant women nationwide in Sweden, with comprehensive data collected on various pregnancy outcomes, allowing for robust statistical analyses. Faecal microbiome sampling at two time points, combined with longitudinal data collection across Sweden, makes the population fairly representative of the general population, although some selection bias is always present in volunteer-based cohorts. The use of advanced, cutting-edge sequencing technology for metagenomic analyses of bacterial DNA and its functions will help us understand the significance and potential role of microbiomes in various outcomes. Furthermore, Sweden’s health registries, which are highly complete and up to date, allow us to cross-reference and minimize biases stemming from incorrectly self-reported data. Finally, using both Swedish and English questionnaires and participant information enabled a broader recruitment.

However, home-based sampling excludes additional informative samples, such as blood samples. The inclusion of patients occurred during pregnancy; therefore, pre-pregnancy microbiome samples were not collected. It may be essential to have pre-pregnancy data to facilitate screening before pregnancy, allowing for comparison of the eventual microbiome changes that occur during pregnancy. Another limitation is that the questionnaires included limited information on diet, sleep and physical activity. As in most studies, there is an over-representation of participants from higher socioeconomic backgrounds and those living in large cities, which may limit the generalizability of the findings to a more diverse population. In particular, while participants in the study were mostly living in larger cities, the highest obesity rates in Sweden are found in smaller towns and villages [51].

While our data conclusively demonstrates differences in the faecal microbiome of lean and obese pregnant women, future work will need to assess whether these differences can be causally linked to various pregnancy outcomes. If so, interventions and monitoring strategies may need to be adapted for obese women, whose baseline microbiome is already distinct from the lean population.

## Conclusion

The faecal microbiomes of one thousand lean and obese women in Sweden change throughout pregnancy, but crucial differences between the groups persist and deepen. Crucially, there is an over-abundance of opportunistic pathogens in the microbiome of obese pregnant women in the third trimester (around week 30). Gut microbiome composition may be one factor modulating the differential risk of various pregnancy complications between lean and obese individuals. If microbiome-based screening can be utilized, or if microbiome-altering approaches become more widely available to obese patients during pregnancy, it could profoundly alter the risk profile for a large portion of pregnancies.

## Supplementary Information

Supplementary Material 1.

Supplementary Material 2.

## Data Availability

The metagenomic sequences, along with selected metadata variables, have been submitted to ENA (project number PRJEB81814).
