# Supplementary material for: Differences in the faecal microbiome of obese and non-obese pregnant women: a matched cohort study in Sweden
Source: BMC Microbiol. 2025 Nov 15;25:750. doi: 10.1186/s12866-025-04473-8 (PMC12619464; doi:10.1186/s12866-025-04473-8)
Supplement: Supplementary file 1 — Supplementary Material 1. [file 12866_2025_4473_MOESM1_ESM.docx]

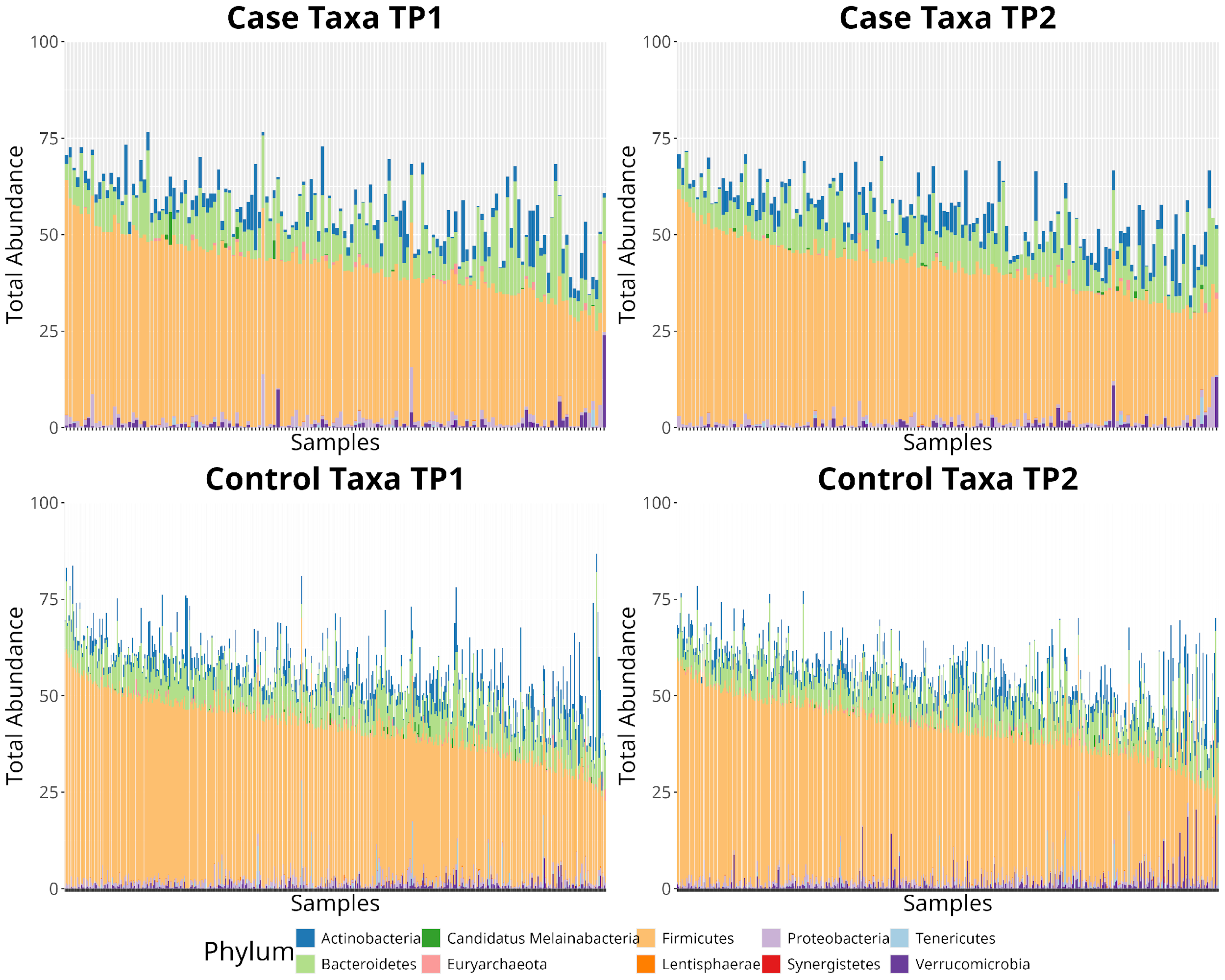


***Figure S1:*** Stacked bar plots of the phylum-level microbial compositions in Obese and Lean groups at both time points.


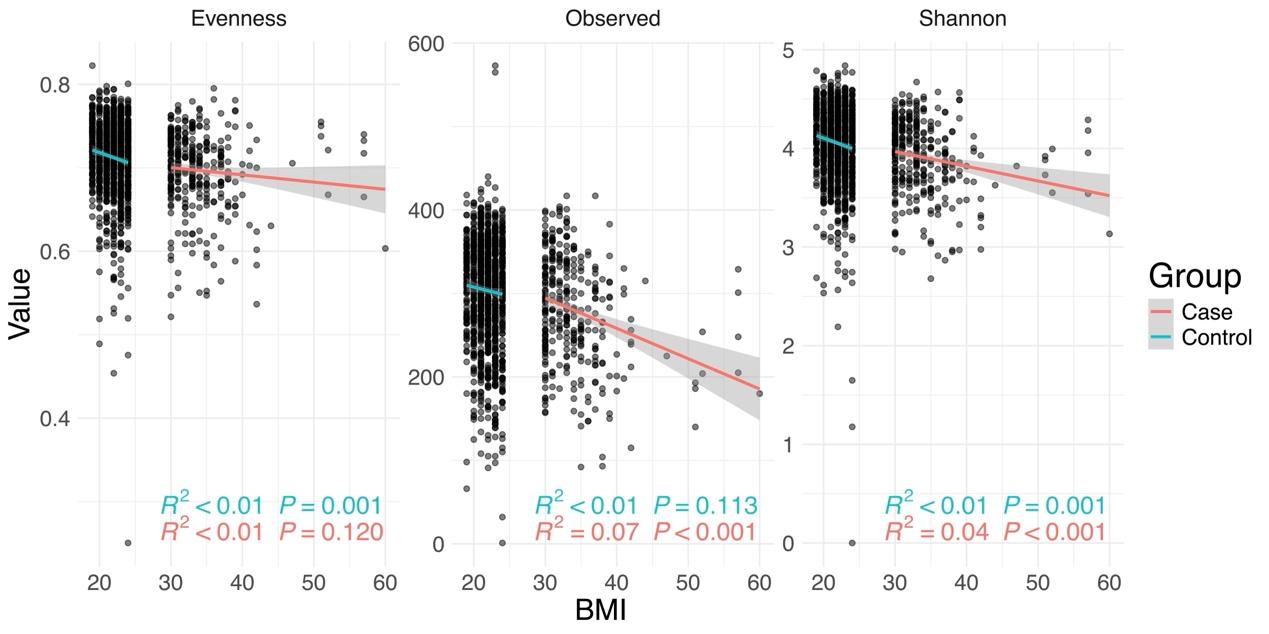


***Figure S2: Scatter plots showing the negative association between BMI and α-diversity.*** In each group, association was estimated by fitting a linear model between the BMI and values of each α-diversity metric, where R^2^ and p values were estimated. Red: linear model, corresponding R2 and p values in the Case group. Blue: linear model, corresponding R2 and p values in the Control group.

***
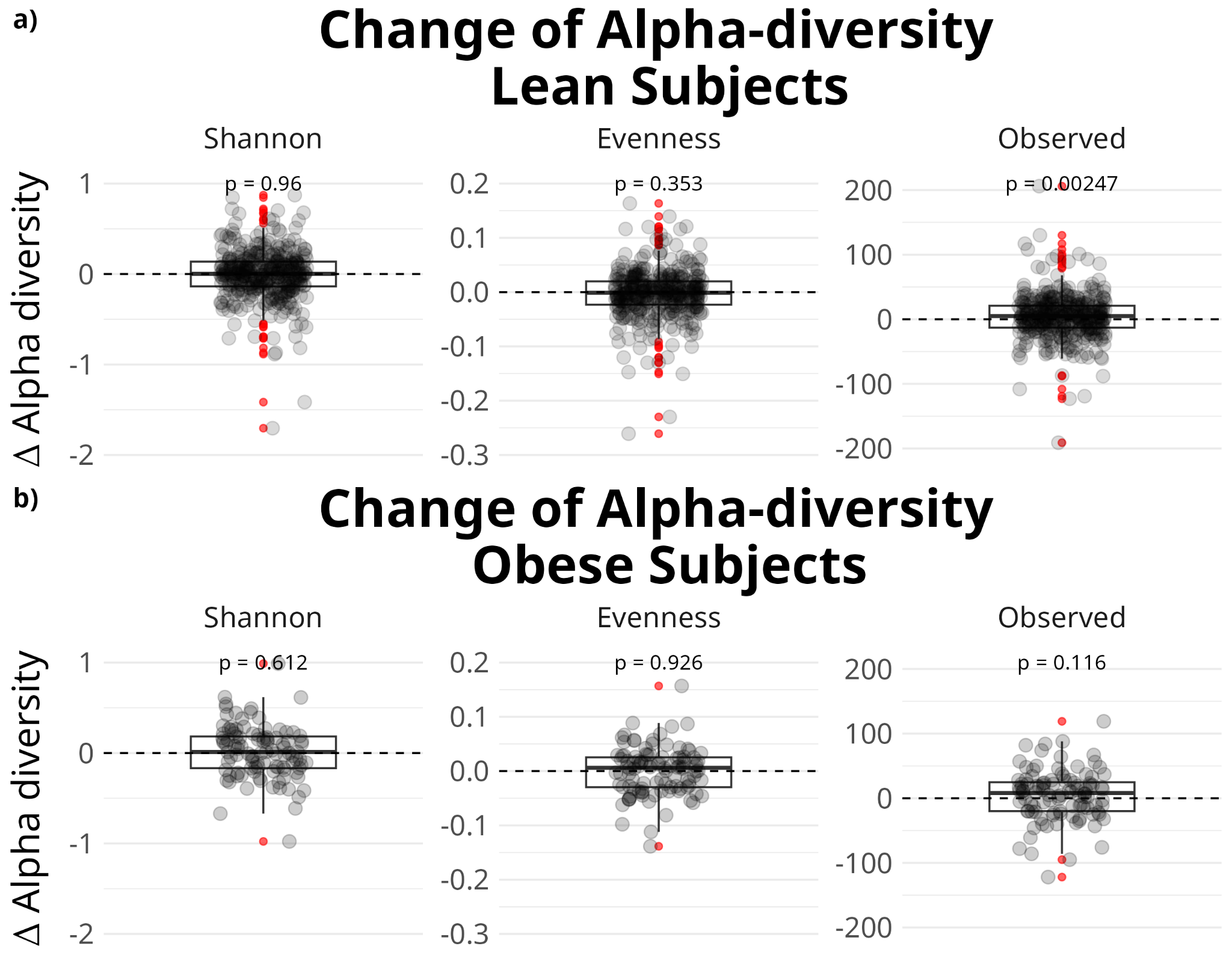
***

***Figure S3:*** ***Species richness is significantly higher at time-point 2 for lean subjects. While the same trend is observed for the obese, it is not significant.*** a) Change of alpha diversity within lean subjects from time point 1 to time point 2. b) Change of alpha diversity within obese subjects from time point 1 to time point 2.


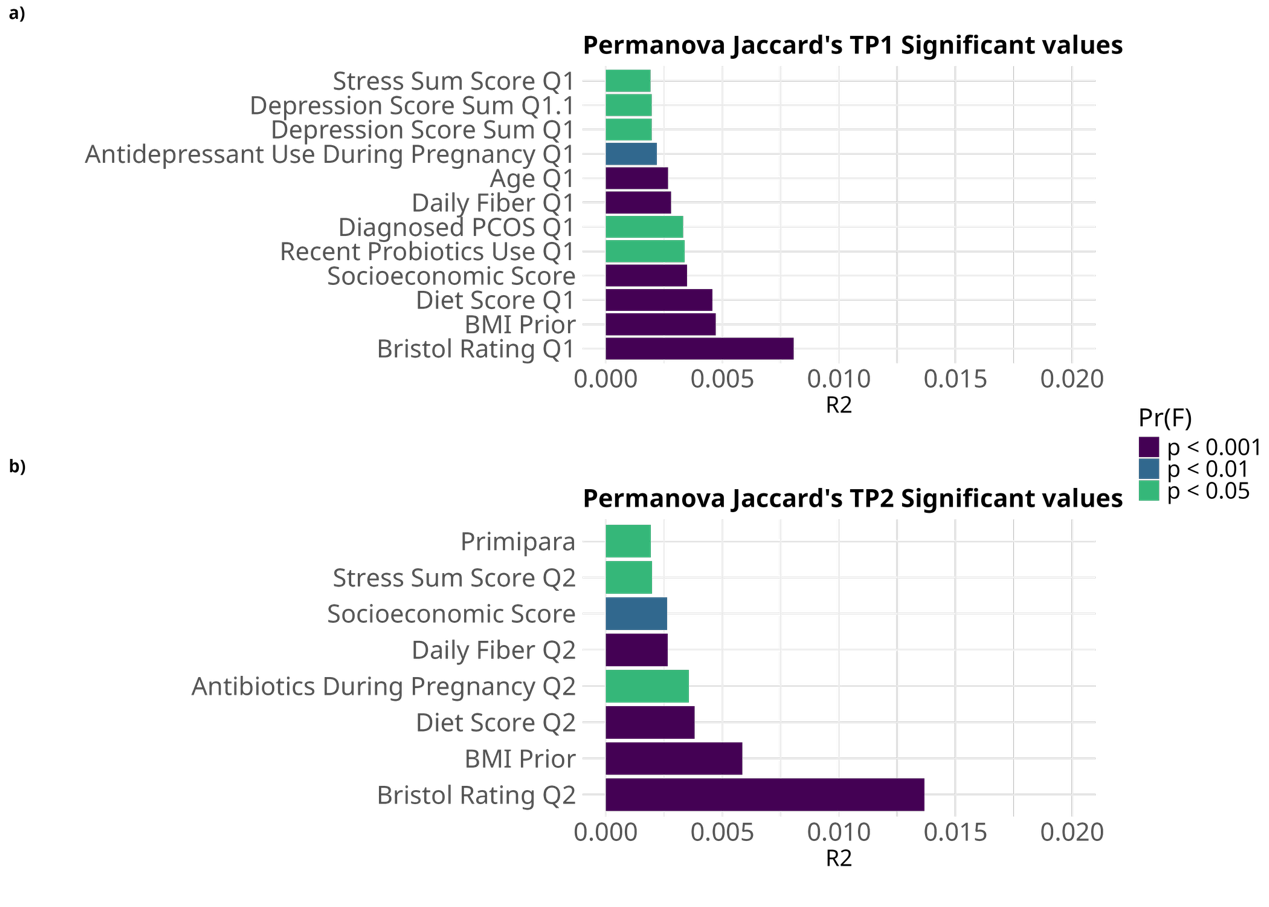


***Figure S4:* Bristol stool form scale, BMI, antibiotics and probiotics are major contributors to differences between samples at both time points**

Permanova R² (length of the bar) and p-value (color scale) based on Jaccard distance at early and late pregnancy. Q1: questionnaire 1 (collected 1-2 weeks before sample 1). Q2: questionnaire 2 (collected 1-2 weeks before sample 2).


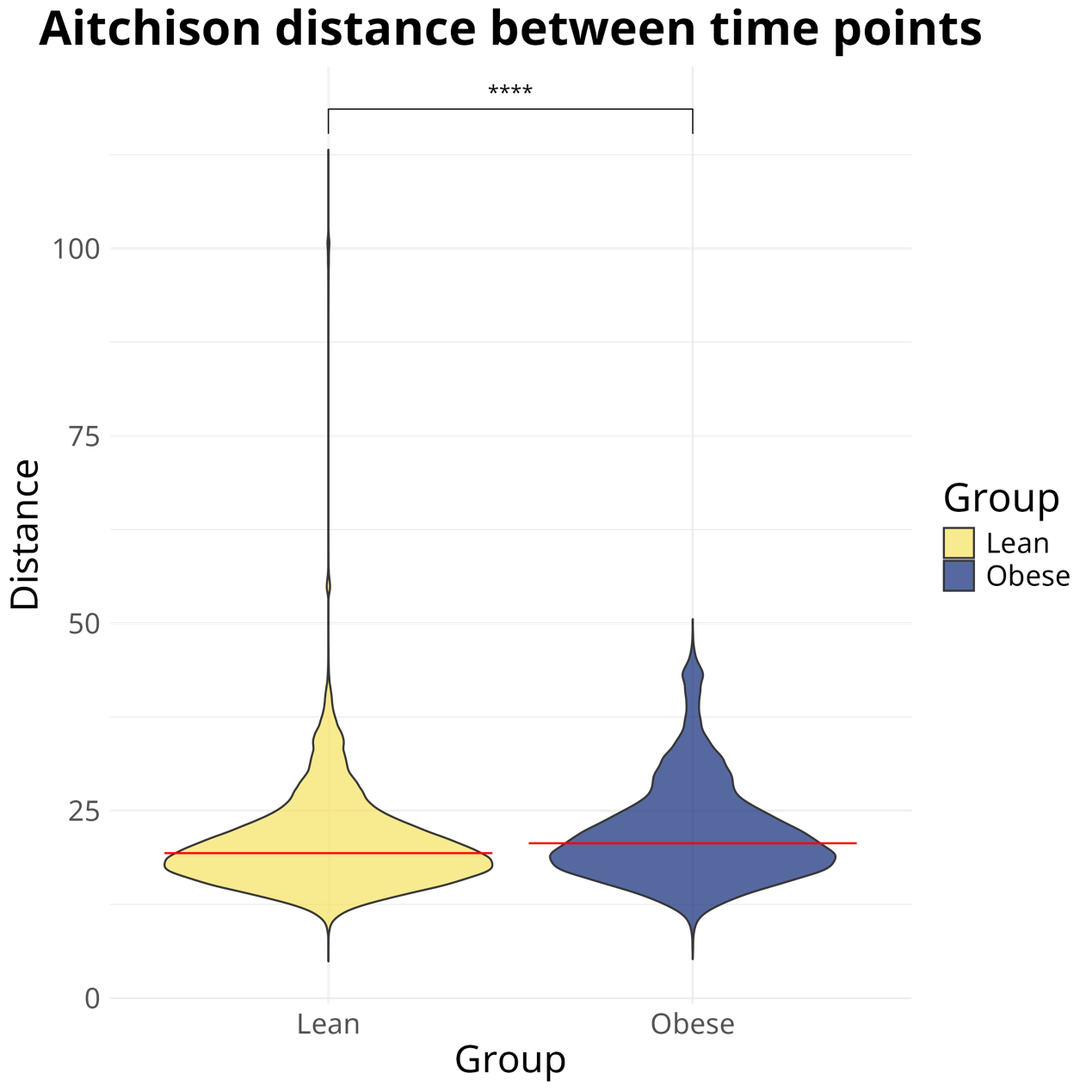
 ***Figure S5: The similarity of gut microbial composition between early and late pregnancy in lean individuals was significantly higher than individuals from the obese group***. For each individual, the Aitchison distance between samples collected at Time point 1 and Time point 2 was calculated, and these distances were compared between the lean and obese groups.


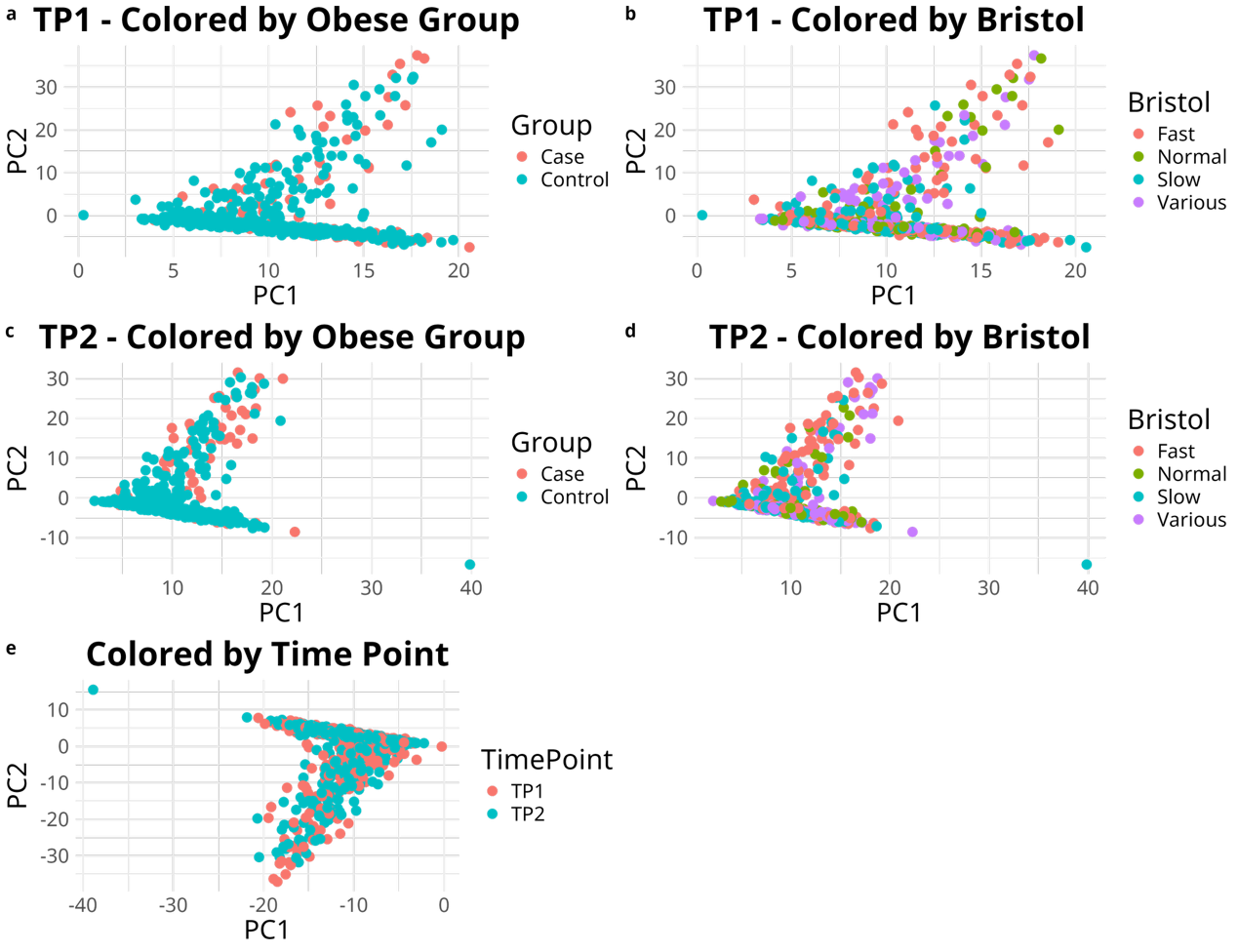


***Figure S6: PCA plots showing relationships among samples colored by different groups.*** a) Samples from lean vs. obese individuals at Time point 1. b) Samples from individuals with different stool transit times at Time point 1. c) Samples from lean vs. obese individuals at Time point 2. d) Samples from individuals with different stool transit times at Time point 2. e) Samples from different time points.


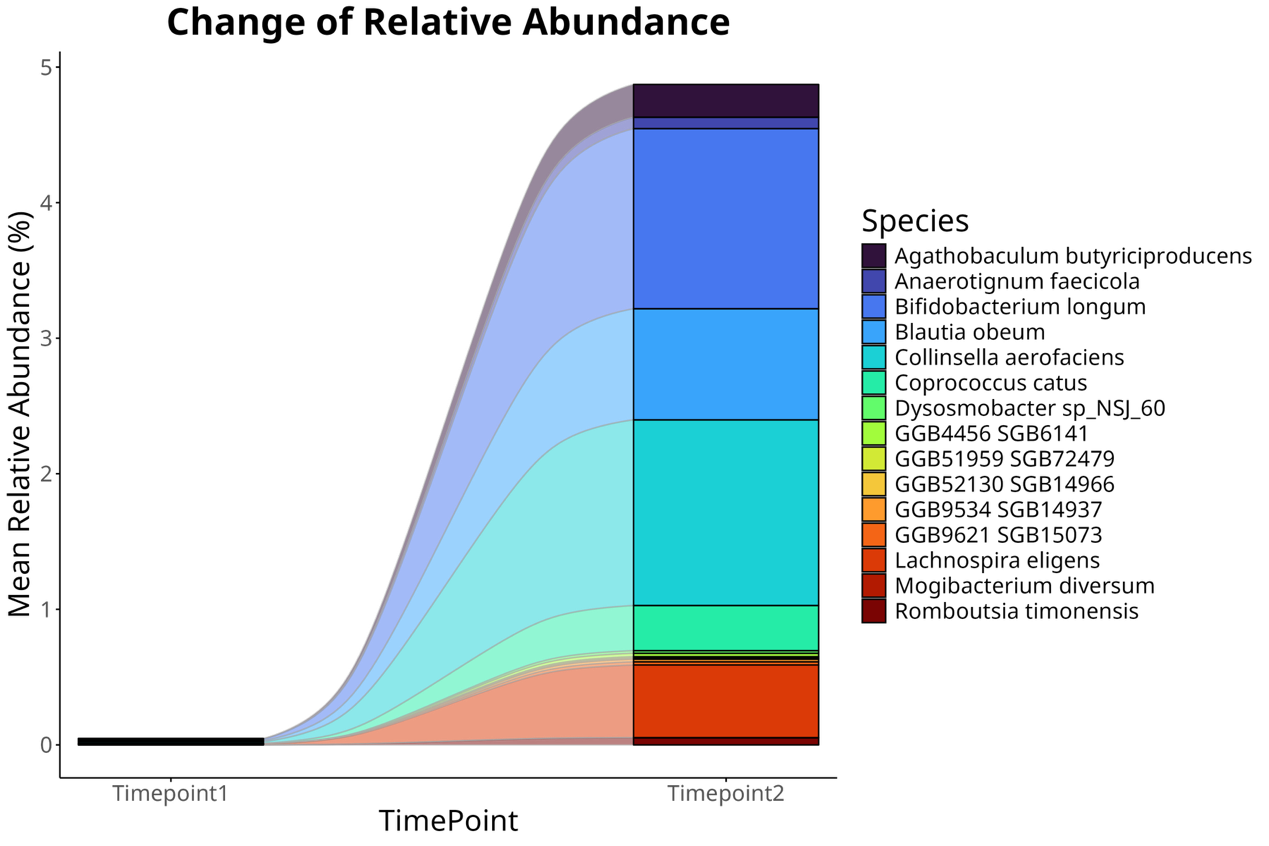


***Figure S7: Bifidobacterium longum and Collinsella aerofaciens were found to be the top two significantly enriched species at time point 2 compared with time point1*.** Stacked bar plot showing changes in the relative abundance of the 15 species identified by differential abundance analysis as significantly more abundant at time point 2 compared with time point 1.
